# Spike-Specific Memory B Cell Response in Hematopoietic Cell Transplantation Recipients following Multiple mRNA-1273 Vaccinations: A Longitudinal Observational Study

**DOI:** 10.3390/vaccines12040368

**Published:** 2024-03-29

**Authors:** Elena Pettini, Annalisa Ciabattini, Fabio Fiorino, Jacopo Polvere, Gabiria Pastore, Monica Tozzi, Francesca Montagnani, Giuseppe Marotta, Alessandro Bucalossi, Donata Medaglini

**Affiliations:** 1 Laboratory of Molecular Microbiology and Biotechnology, Department of Medical Biotechnologies, University of Siena, 53100 Siena, Italy; annalisa.ciabattini@unisi.it (A.C.); fiorino4@unisi.it (F.F.); jacopo.polvere@student.unisi.it (J.P.); gabiria.pastore@unisi.it (G.P.); donata.medaglini@unisi.it (D.M.); 2 Department of Medicine and Surgery, LUM University “Giuseppe Degennaro”, 70010 Bari, Italy; 3 Cellular Therapy Unit, Department of Innovation, Experimentation, Clinical and Translational Research, University Hospital of Siena, 53100 Siena, Italy; m.tozzi@ao-siena.toscana.it (M.T.); g.marotta@ao-siena.toscana.it (G.M.); alessandro.bucalossi@ao-siena.toscana.it (A.B.); 4 Department of Medical Biotechnologies, University of Siena, 53100 Siena, Italy; francesca.montagnani@unisi.it; 5 Infectious and Tropical Diseases Unit, Department of Medical Sciences, University Hospital of Siena, 53100 Siena, Italy

**Keywords:** mRNA-based vaccine, allogeneic hematopoietic cell transplantation recipients, vaccination, SARS-CoV-2, spike-specific memory B cells

## Abstract

Preventing SARS-CoV-2 infection is of utmost importance in allogeneic hematopoietic cell transplantation patients (allo-HCT), given their heightened susceptibility to adverse outcomes associated with SARS-CoV-2 infection. However, limited data are available regarding the immune response to COVID-19 vaccines in these subjects, particularly concerning the generation and persistence of spike-specific memory response. Here, we analyzed the spike-specific memory B cells in a cohort of allo-HCT recipients vaccinated with multiple doses of the mRNA-1273 vaccine and monitored the spike-specific antibody response from baseline up to one month after the fourth dose. After the primary vaccine series, the frequency of spike-specific B cells, detected within the pool of Ig-switched CD19+ cells, significantly increased. The booster dose further induced a significant expansion, reaching up to 0.28% of spike-specific B cells. The kinetics of this expansion were slower in the allo-HCT recipients compared to healthy controls. Spike-specific IgG and ACE2/RBD binding inhibition activity were observed in 80% of the allo-HCT recipients after the first two doses, with a significant increase after the third and fourth booster doses, including in the subjects who did not respond to the primary vaccine series. Additionally, 87% of the allo-HCT recipients exhibited positive cross-inhibition activity against the BA.1 variant. Our findings provide evidence that allo-HCT recipients need repeated doses of the mRNA-1273 vaccine to induceSARS-CoV-2 specific immune response similar to that observed in healthy individuals. This is particularly crucial for vulnerable individuals who may exhibit a limited response to the primary series of SARS-CoV-2 vaccination.

## 1. Introduction

Patients undergoing autologous (auto) and allogeneic (allo) hematopoietic cell transplantation (HCT) for hematologic malignancies have a higher risk of adverse outcomes linked to severe acute respiratory syndrome coronavirus 2 (SARS-CoV-2) infection compared to the general population [1]. SARS-CoV-2 infection in allo-HCT recipients has shown an associated mortality rate of around 20% compared to 1.8% observed in the overall population of the United States [1,2]. Additionally, HCT recipients tend to have a prolonged shedding of SARS-CoV-2, leading to a prolonged duration of symptoms and fostering the emergence of highly mutated viruses [3]. Therefore, vaccination has been strongly recommended and prioritized for these fragile patients. However, HCT recipients commonly display variable and sometimes suboptimal immune responses to vaccines when compared to healthy individuals [4,5]. This period of dysfunctional immunity linked to a heightened risk of infections can be attributed to the diverse underlying pathology that impairs their immune system functionality and to the administered therapies that leave them severely immunocompromised for extended periods of time. [1,2,6]. Previous research on influenza and pneumococcus vaccines has revealed a diminished antibody response in allo-HCT recipients when compared to healthy individuals [7,8,9]. However, despite this weaker response, influenza vaccination has shown clinical benefits in allo-HCT recipients [10]. With the introduction of novel mRNA vaccines, the FDA and EMA approved their use in vulnerable populations in 2020. Subsequently, the Centers for Disease Control and Prevention adjusted the initial two-dose primary series, strongly promoting the administration of booster doses of mRNA vaccine for vulnerable populations [11]. While SARS-CoV-2 mRNA vaccination has been demonstrated to generally induce elevated levels of antibodies in the majority of HCT recipients after two vaccine doses, there are cases where some patients do not develop a substantial immune response to SARS-CoV-2 vaccination [12]. Recent studies have shown that administering a booster dose of mRNA SARS-CoV-2 vaccine significantly improves spike-specific humoral immunity in HCT recipients who have shown a limited response to the initial COVID-19 vaccine series [13]. This finding aligns with similar observations made in patients who have undergone solid-organ transplantation, highlighting the significance of an extra vaccine dose for individuals who may have achieved suboptimal humoral immune responses following the primary series of COVID-19 vaccination [14,15]. While the majority of immunogenicity studies on COVID-19 vaccination have focused on analyzing the induction and durability of spike-specific antibody responses, limited data are available on the induction and duration of memory B cell response. Understanding the persistence of memory B cells is crucial as they play a crucial role in the immune system’s capability to recognize and rapidly respond to pathogen re-exposure. In this study, we conducted a longitudinal analysis to assess the production of spike-specific antibodies and their capacity to bind the spike protein, and, most importantly, we examined the spike-specific B cell response in allo-HCT recipients who received a four-dose regimen of the mRNA-1273 vaccine. The analysis was performed from the baseline up to one month after the fourth dose, allowing us to closely examine the immune response over time and evaluate the induction and maintenance of spike-specific memory B cells in this fragile population.

## 2. Materials and Methods

### 2.1. Study Participants

**Figure 1:**
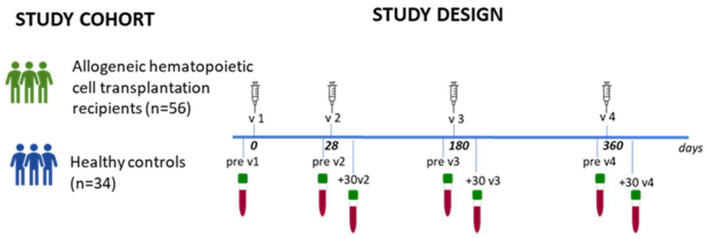
Schematic representation of the study participants and design. Allogeneic hematopoietic cell transplantation (allo-HCT) recipients (56 subjects) and healthy controls (HCs; 34 subjects) were vaccinated with up to four doses of mRNA-1273 Moderna at day 0, 28, 180, and 360. Blood samples were collected at pre v1 (day 0, baseline), pre v2, +30 v2 (before and 1 month post second dose), pre v3, +30 v3 (before and 1 month post third dose), and pre v4, +30 v4 (before and 1 month post fourth dose). Plasma samples were analyzed for spike-specific IgG and ACE2/RBD inhibition, while peripheral blood mononuclear cells (PBMCs) were examined for spike-specific memory B cell response.

Blood samples were obtained from 56 adult allogeneic hematopoietic cell transplantation recipients (allo-HCT) and from 34 healthy controls (HCs) who received up to four doses of the mRNA-1273 (Spikevax, Moderna, Cambridge, MA, USA) vaccine. Among the allo-HCTs, 89% completed the third dose, and of these, 80% also completed the fourth dose. Among the HCs, all completed the third dose, while only 52% proceeded to the fourth dose. According to national schedules, the first and second vaccine doses were administered 4 weeks apart. Subsequently, the third and the fourth doses were administered about 6 months after the previous ones (Figure 1). History of SARS-CoV-2 infection before vaccination served as a criterion for exclusion. Participants were asked to respond to a survey to gather information on infection contracted from the first vaccine administration up to July 2022, including the date of the positive antigenic or molecular tests, performed on self-administered or professional-collected nasopharyngeal swabs. Self-reported infected subjects were symptomatic or were in contact with infected persons and did not include people who could have contracted the infection in an asymptomatic way. All participants provided written informed consent before joining the study. Enrollment took place at the Cellular Therapy Unit, Azienda Ospedaliera Universitaria Senese (Siena, Italy). The study adhered to all pertinent ethical regulations, and the protocol received approval from the local Ethical Committee for Clinical experimentation of Regione Toscana Area Vasta Sud Est (CEAVSE), protocol code 19479 PATOVAC v1.0 of 3 March 2021, approved on 15 March 2021, and protocol code 18869 IMMUNO_COV v1.0 of 18 November 2020, approved on 21 December 2020. The study protocol was designed during the initial pandemic phase when no data on immunogenicity and effectiveness of mRNA vaccine in allo-HCT recipients were available. Therefore, the study was conceived as a pilot study without the feasibility of calculating a sample size.

### 2.2. Plasma and Peripheral Blood Mononuclear Cells Isolation

Blood samples were collected at baseline (pre v1), before the second, third, and fourth dose (pre v2, pre v3, and pre v4, respectively), and 30 days after the second, third, and fourth dose (+30 v2, +30 v3, and +30 v4, respectively, as reported in Figure 1). PBMCs were isolated by density-gradient sedimentation and cryopreserved as previously described [16].

### 2.3. ELISA and ACE2/RBD Inhibition Assays

The production of spike-specific IgG was assessed using recombinant SARS-CoV-2 Spike S1 + S2 ECD (1 μg/mL protein; Sino Biological, Eschborn, Germany) as a coating following previously described procedures [16]. WHO international positive (NIBSC 20/150) and negative (NIBSC 20/142) controls were included in duplicate on each plate as internal controls to guarantee assay reproducibility. ACE2/RBD inhibition was assessed using a SARS-CoV-2 surrogate virus neutralization test (sVNT) kit (cPass™, Genscript, Rijswijk, The Netherlands) according to the manufacturer’s protocol and as previously detailed [16]. Inhibition values ≥ 30% were considered positive, while values < 30% were considered negative, following the manufacturer’s guidelines.

### 2.4. Multiparametric Flow Cytometry

To analyze B cell populations and to identify SARS-CoV-2-specific B cells within PBMC by flow cytometry, a multi-color panel was developed. PBMCs were incubated with the BD human Fc block (BD Biosciences, Aalst, Belgium) for 10 min at RT. The cells were then stained with the SARS-CoV-2 spike full protein ECD-His recombinant biotinylated-protein (25 µg/mL, Sino Biological) in a staining buffer [PBS, 0.5% bovine serum albumin (BSA) and 2 mM EDTA, all from Sigma-Aldrich, St. Louis, MO, USA/Burlington, MA, USA] for 30 min at 4 °C, followed by staining with FITC- and Brillant-Violet-421-conjugated streptavidin for an additional 30 min at 4 °C. Subsequently, the cells were stained for 30 min at 4 °C using the subsequent antibody mixture, comprising CD3-PECy 7 (clone SK7), CD56-PECy7 (clone B159), CD14-PECy7 (clone M5E2), CD19-BUV395 (clone SJ25C1), IgM-BV605 (clone G20-127) and IgD-PE (clone IA6-2) (all from BD Biosciences, Belgium). The cells were then labeled with live/dead FSV780 following the manufacturer’s instructions (BD Biosciences). Finally, the cells were fixed using a BD fixation solution (BD Biosciences) and analyzed with an SO LSRFortessa X20 flow cytometer (BD Biosciences). Data analysis was conducted using FlowJo v10 (TreeStar, Ashland, OR, USA).

### 2.5. Statistical Analysis

Descriptive statistics, including numbers, medians, interquartile ranges, and frequencies, were employed to depict the baseline characteristics of the patients. The age distribution and gender ratio were assessed between groups using the non-parametric Mann–Whitney test and Fisher’s exact test, respectively. The Kruskal–Wallis test, followed by Dunn’s post-test for multiple comparisons, was utilized to evaluate the statistical differences of the ELISA titers as well as the percentages of ACE2/RBD inhibition. The Mann–Whitney test was applied to evaluate the statistical differences in spike-specific IgG between HC and HCT for each time point and in ACE2/RBD inhibition percentages. The Mann–Whitney test was also used to analyze the differences in spike-specific B cell frequencies. The potential association between the clinical and demographic variables of allo-HCT and log-transformed spike-specific IgG ELISA titers, detected at +30 v3 and +30 v4, were investigated by a multiple linear regression analysis. Analyses were performed using GraphPad Prism v10 (GraphPad Software, San Diego, CA, USA). For all statistical analyses, the significance level was set to 0.05.

## 3. Results

The induction of spike-specific immune responses was monitored in a cohort of 56 allo-HCT recipients and 34 HCs after the second, third, and fourth doses of the mRNA-1273 vaccine (Figure 1). As described in Table 1, the median time from transplantation to vaccination was 6.4 years (range 0.2–18.9 years), and at the time of the first vaccination, 11 patients (20%) were still undergoing immunosuppressive therapy, and no relevant changes in treatment were observed during the study. Primary pathologies were mainly acute myeloid leukemia (52%) and acute lymphoblastic leukemia (18%). 

### 3.1. Spike-Specific Antibody Response and ACE2/RBD Inhibition Binding Activity

**Figure 2:**
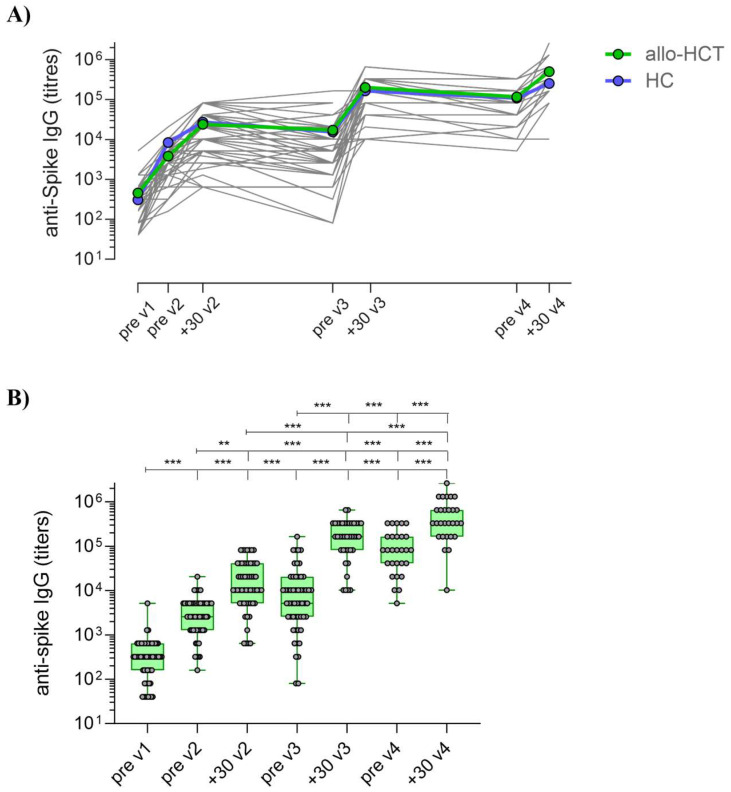
Spike-specific IgG production against SARS-CoV-2 in allo-HCT recipients. (**A**) The anti-spike IgG titers were assessed over time using ELISA, with sampling conducted at baseline (prior to the first dose, pre v1), before the second dose (pre v2), 30 days after the second dose (+30 v2), before the third dose (pre v3), 30 days after the third dose (+30 v3), before the fourth dose (pre v4), and 30 days after the fourth dose (+30 v4). Data are reported in grey for individual allo-HCT patients and in green the mean value. Healthy subjects were included as controls (HCs), and the mean value is reported in blue. (**B**) Spike-specific IgG were evaluated in individual allo-HCT recipients at different time points. Data are presented in a box-and-whiskers diagram illustrating the minimum and maximum values of the entire dataset. Antibody end-point titers are indicated as the reciprocal of the sample dilution, reporting double the background OD value. Kruskal–Wallis’ test, followed by Dunn’s post-test for multiple comparisons, was employed to assess statistical differences between groups; ** *p* ≤ 0.01; *** *p* ≤ 0.001.

The spike-specific IgG response induced by SARS-CoV-2 mRNA vaccination in the allo-HCT recipients and HCs was assessed by ELISA on plasma samples collected before each vaccine administration and 30 days after the second (+30 v2), third (+30 v3), and fourth (+30 v4) vaccine dose. In Figure 2A, the kinetics of spike-specific IgG in each allo-HCT recipient is shown in grey, while their mean value is represented in green, and the mean value of anti-spike IgG in the HCs is reported in blue. As is evident from the overlapping spike-specific IgG curves (Figure 2A), there was no significant difference (assessed by Mann–Whitney test) in the humoral response triggered by the mRNA-1273 vaccine between the allo-HCT recipients and HCs (Figure 2A). The kinetics of spike-specific IgG in each HC is reported in Supplementary Figure S1.

At the baseline (pre v1), the anti-spike IgG geometric mean titer (GMT) was 230 [95% confidence interval (CI): 260 to 620; titers range: 40–640], and significantly increased 4 weeks after the first dose (pre v2), reaching a GMT of 2560 [95% CI: 2942 to 4811; titers range: 160–20,480; *p* ≤ 0.001; Figure 2A,B]. At the same time point, HCs developed spike-specific IgG with a GMT of 8320 (Figure 2A). The second dose boosted the anti-spike IgG in allo-HCT recipients, as observed 30 days later (+30 v2), with a GMT of 12,638 [95% CI: 17,487 to 31,199; titers range: 640–81,920; *p* ≤ 0.01; Figure 2A,B]. Six months after the second dose (pre v3), the levels of anti-spike IgG slowly decreased, with a GMT of 5747 [95% CI: 9212 to 25,402; titers range: 80–163,840; Figure 2A,B]. The third vaccine dose significantly enhanced antibody levels in the allo-HCT recipients, reaching after 30 days (+30 v3) a GMT of 132,372 [95% CI: 154,059 to 256,591; titers range: 10,240–655,360; *p* ≤ 0.001; Figure 2A,B]. A not-significant decrease was observed six months later (pre v4), with a GMT of 70,613 [95% CI: 75,572 to 161,776; titers range: 5120–327,680; Figure 2A,B]. The fourth vaccine dose robustly increased the IgG levels, with a GMT of 319,374 [95% CI: 327,605 to 82,818; titers range: 10,240–1,310,720; Figure 2A,B], in the allo-HCT recipients (+30 v4). No statistically significant differences, evaluated by the unpaired Mann–Whitney test, were observed in the spike-specific humoral response between the allo-HCT recipients and HCs for all time points analyzed, as shown by the overlapping green and blue lines in Figure 2A, highlighting a similar vaccine-induced antibody response for both groups. 

We also investigated clinical and demographic variables associated with spike-specific IgG titers at +30 v3 and +30 v4 by multiple linear regression analysis (Supplementary Table S1). We observed a significant negative association of IgG titers with ongoing immunosuppressive therapy (*p* = 0.044) after the third dose. Other variables (age, male gender, years post transplantation, and chronic GVHD) had a negligible negative impact on the IgG response (*p* > 0.05). Interestingly, after the fourth, dose no significant influence of clinical and demographic variables on IgG response was registered, remarking that multiple booster doses induce a strong humoral response even in patients with immunosuppressive therapy. 

**Figure 3:**
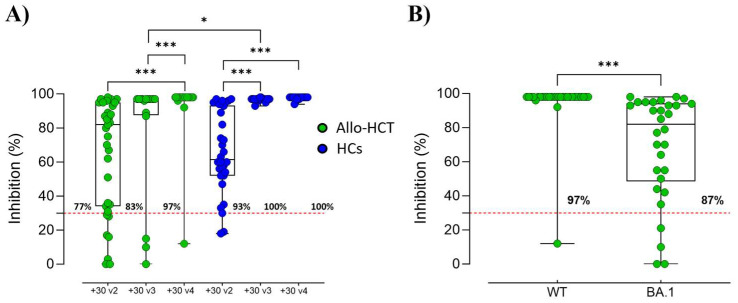
ACE2/RBD binding inhibition following SARS-CoV-2 mRNA vaccination identified in plasma of allo-HCT recipients and HCs. (**A**) ACE2/RBD inhibition for wild-type SARS-CoV-2 was assessed at +30 v2, +30 v3, and +30 v4. A threshold (indicated by dotted red line) was set at 30% inhibition percentage to differentiate between positive and negative samples. Kruskal–Wallis’ test, followed by Dunn’s post-test for multiple comparisons, was employed to evaluate statistical differences between time points within allo-HCT recipients (green dots) and in HCs (blue dots). Mann–Whitney’s test was used for assessing statistical differences within HC cohort and between the same time points of the two cohorts. * *p* ≤ 0.05; *** *p* ≤ 0.001. (**B**) Comparison of ACE/RBD inhibition for wild-type (WT) and Omicron/BA.1 (BA.1) variants were assessed at +30 v4 in allo-HCT. Mann–Whitney’s test was used for assessing statistical differences between responses against the two variants. *** *p* ≤ 0.001.

To further investigate the role of the anti-spike IgG induced by vaccination, a surrogate virus neutralization assay was employed to assess the capacity of vaccine-induced antibodies to inhibit the ACE2/RBD interaction and its persistence at +30 v2, +30 v3, and +30 v4 (Figure 3A). A general increase in the mean inhibition ± standard deviation values was observed for the allo-HCT recipients throughout the time of the study. The fourth dose significantly increased the inhibition percentages compared to the levels observed after the second (94.87% ± 15.69% vs. 65.43% ± 33.30%; *p* ≤ 0.001) and third doses (94.87% ± 15.69% vs. 79.19% ± 35.39%; *p* ≤ 0.001). The percentages of responders gradually increased after multiple doses, with 77% at +30 v2, 83% at +30 v3, and +97% at +30 v4. No significant differences were observed when comparing the mean inhibition at +30 v2 between the allo-HCT recipients and HCs (65.43% ± 33.30% vs. 65.70% ± 24.17%; *p* = 0.645), while a significantly higher response was observed in the controls at +30 v3 (79.19% ± 35.39% vs. 96.78% ± 0.998%; *p* ≤ 0.05) (Figure 3A). In the HCs, the percentages of responders were high already after two doses, with 93% at +30 v2 and reaching and maintaining 100% at +30 v3 and +30 v4.

The ability of vaccine-induced IgG antibodies of inhibiting ACE2/RBD binding, in the allo-HCT recipients was also evaluated after the fourth dose (+30 v4) using the RBD protein from the Omicron variant (BA.1) and was compared to wild-type SARS-CoV-2 RBD (Figure 3B). The binding inhibition when using the BA.1 RBD was significantly lower (69.07% ± 30.98% vs. 94.87% ± 15.69%, *p* ≤ 0.001) with respect to the wild-type RBD. However, it is important to note that one month after the fourth vaccine dose, 87% of the allo-HCT recipients exhibited antibodies with the ability to inhibit ACE2/RBD of the BA.1 variant binding.

### 3.2. Spike-Specific Memory B Cells following SARS-CoV-2 mRNA Vaccination in Allo-HCT Recipients

**Figure 4:**
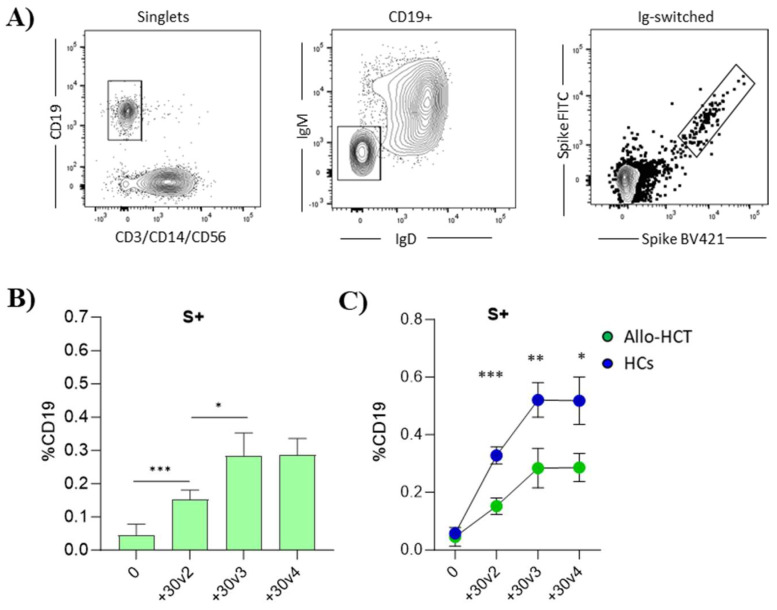
Spike-specific memory B cells. (**A**) Gating strategy for the identification of S+ IgG–switched B cells. From left to right: B cells were gated as CD19+, CD3− CD14− CD56− cells. Within the B cells gate, spike-specific B cells were defined as Ig-switched B cells (IgM− IgD−) and double-positive for S FITC and S BV421 (S+ B cells). (**B**) Bar graphs show the percentages of S+ B cells at baseline and 30 days after the second, third, and fourth mRNA-1273 vaccine doses in allo-HCT recipients. (**C**) Frequencies of S+ B cells at different time points in allo-HCT recipients (green dots) compared to HCs (blue dots). Mann–Whitney’s test, followed by Dunn’s post-test for multiple comparative tests, was used for assessing statistical differences between cell frequency at different time points. * *p* ≤ 0.05, ** *p* ≤ 0.01, *** *p* ≤ 0.001.

Eliciting broad reactive memory B cells is a key aspect in vaccine development, since memory B cells take an important part in the immune response, acting as a secondary defense line and triggering a rapid increase in Ab-secreting plasma cells in case of pathogen exposure ([17]). Here, we profiled the immune memory elicited by SARS-CoV-2 vaccination and its persistence by analyzing PBMCs samples collected 30 days after the second, third, and fourth vaccine dose (+30 v2, +30 v3, and +30 v4) in the allo-HCT recipients and HCs by multiparametric flow cytometry (Figure 4). Circulating SARS-CoV-2-specific B cells were identified by employing the full-spike protein with two distinct fluorescent probes according to the expression of CD19, IgD and IgM molecules. Spike-specific B cells, referred to as S+ B cells, were identified within the pool of Ig-switched CD19^+^ cells using the gating strategy outlined in Figure 4A. The frequency of antigen-specific memory B cells in the allo-HCT subjects increased already after the second dose, as evidenced by the statistically significant rise in S+ B cells compared to the baseline (0.15% at +30v2 versus 0.04% at day 0; *p* ≤ 0.001) (Figure 4B). Furthermore, a further significant increase was observed after the third dose, with the detection of 0.28% S+ B cells (*p* ≤ 0.05), while no further increase was observed after the fourth vaccine dose administration. It is worth noting that the trend of the increase in spike-specific S+ B cells was similar in the allo-HCT subjects and controls, but the frequencies of S+ B cells in the allo-HCT subjects at +30 v2, +30 v3, and +30 v4 were consistently significantly lower than in the healthy individuals (0.15% vs. 0.32%, 0.28% vs. 0.52%, and 0.28% vs. 0.51%, respectively; *p* ≤ 0.001, *p* ≤ 0.01, *p* ≤ 0.05; Figure 4C).

Overall, these data suggest that repeated administrations of the mRNA-1273 vaccine induce a pool of memory S+ B cells in allo-HCT subjects, with a similar trend to that observed in healthy individuals. The statistically lower frequencies of S+ B cells in the allo-HCT subjects may be attributed to the close administration of vaccine doses in these fragile individuals, not allowing the immune response activated after one vaccination to wane before the next dose. A vaccination schedule with more spaced doses, as occurs in healthy subjects, could be beneficial in adopting in the perspective of increasing the pool of memory S+ B cells in fragile individuals.

## 4. Discussion

In this study, we examined the immune responses triggered by multiple booster doses of RNA-1273 in 56 allo-HCT recipients and 34 HCs. We focused on analyzing the development of spike-specific memory B cells and assessing the quantity and functionality of spike-specific antibodies in allo-HCT recipients up to one month after the fourth vaccine dose. The spike-specific memory B cells showed a significant increase in the allo-HCT recipients after repeated RNA-1273 booster doses, exhibiting slower kinetics compared to the HCs. Additionally, we demonstrated the concurrent production of spike-specific IgG antibodies in the allo-HCT recipients, comparable to that observed in the HCs. Notably, in 87% of the allo-HCT recipients, the antibodies were capable of inhibiting the binding of the BA.1 variant to ACE2/RBD, showing potential effectiveness against this variant. A history of allogeneic hematopoietic stem cell transplantation constitutes a significant risk factor for severe COVID-19, characterized by morbidity and mortality rates that can surpass 20%, exceeding those observed in healthy individuals. This heightened risk is primarily attributed to factors such as immune dysregulation, extended periods of immunosuppression, graft-versus-host disease, and the presence of comorbidities [2,18,19]. Although individuals who have recently undergone transplantation may exhibit a diminished response to COVID-19 vaccines, it remains imperative to prevent infections in these patients given the unequivocal benefits of vaccination [20]. Several studies, including our previous work [13], have demonstrated that SARS-CoV-2 mRNA vaccination elicits high levels of antibodies in the majority of HCT recipients after a two-dose primary series. However, it is important to note that some patients do not develop a significant immune response to the primary series of SARS-CoV-2 vaccination [12,13]. Hence, administering additional vaccine doses is essential to effectively enhance the immune system, especially in low responders who may experience weakened or delayed immune priming [12,13,21]. Consequently, there is a need for further immunogenicity data, not only regarding the humoral response but also the memory spike-specific B cell response, to inform decisions about vaccinating vulnerable subjects. We have already analyzed in detail the role as well as the dynamics and magnitude of the spike-specific B cell response in healthy subjects in the context of SARS-CoV-2 vaccination [16,22]. Here, we longitudinally profiled spike-specific antibody production and their functionality and, for the first time, the spike-specific memory B cells induced by SARS-CoV-2 vaccination and their persistence from baseline up to one month after the fourth mRNA-1273 vaccine administration in allo-HCT recipients. The frequency of spike-specific B cells, detected within the pool of Ig-switched CD19+ cells, significantly increased after the second vaccine dose. A further significant expansion was elicited by the booster dose, characterized by lower frequencies of spike-specific B cells and a slower kinetics in the allo-HCT recipients compared to the HCs. To enhance the magnitude of the vaccine-induced immune response, as previously demonstrated in preclinical studies [23], it would probably be useful to increase the time interval between a booster dose and the next one in fragile subjects, allowing the antigen-specific immune response activated by the previous vaccination to wane before the administration of the next one. In terms of kinetics, a slower B cell response compared to that seen in healthy individuals has been also observed in a recent study performed by our group in myelofibrosis patient cohort [24]. Further studies aim to investigate the long-term persistence of vaccine-induced B cells, which play a crucial role in protection against SARS-CoV-2 infection. The antibody response in allo-HCT recipients is influenced by various factors, including the number of CD19+ cells, the timeframe following transplantation, complete remission status, the use of immunosuppressive drugs, and the levels of lymphocyte subpopulations [25]. Recent studies have demonstrated the safety and efficacy of the primary cycle of the mRNA-based SARS-CoV-2 vaccine BNT162b2 in allo-HCT recipients, with approximately 50–80% of subjects generating a protective immune response [7,26,27,28,29]. However, limited data are available regarding neutralizing antibody levels in allo-HCT recipients following SARS-CoV-2 vaccination. It was reported that after two doses of mRNA vaccine, about 50% of allo-HCT recipients had neutralizing antibodies above the positive threshold, which was significantly lower than the neutralizing antibody rates observed in HCs [30]. Here, we observed spike-specific IgG antibody production in 90% of the HCT recipients following the primary series of SARS-CoV-2 vaccination up to six months after the second dose (pre v3), and a robust increase was observed after the third and the fourth doses. No statistically significant differences were observed in the spike-specific humoral response between the allo-HCT recipients and HCs for all the time points analyzed. Despite the increase in antibody titers observed in the allo-HCT recipients following the administration of the fourth dose of the mRNA-1273 vaccine, consistent with findings reported by Mittal and colleagues [31], our allo-HCT cohort exhibited a cumulative 22% incidence of breakthrough SARS-CoV-2 infections up to five months after the fourth vaccine dose administration. At the same time, July 2022, 38% of the HCs declared a SARS-CoV-2 infection. This observation, rather than being attributed to a diverse susceptibility to infection, is likely associated with SARS-CoV-2-preventive behaviors adopted by the allo-HCT patients (e.g., greater social distancing and wearing a face mask in social occasions) and the different time intervals at which the booster vaccine doses were administered in the allo-HCT recipients compared to the HCs. 

Concerning the functionality of the observed spike-specific IgG, the third and fourth doses significantly increased the ACE2/RBD inhibition percentages compared to the levels observed after the second dose, together with a gradual increase in the percentages of responders after multiple doses. ACE2/RBD binding inhibition was observed in most of the allo-HCT recipients, including the 10% of subjects that did not respond to the primary series of SARS-CoV-2 vaccination. In general, patients receiving a hematopoietic stem cell transplant typically undergo a short duration of immunosuppressive treatment. At the conclusion of this treatment, the immune system is considered restored. This is in contrast to solid-organ transplantation recipients, where immunosuppressive treatment continues throughout life. These treatments could impact the immune response to vaccinations and may have an impact on the generation of neutralizing antibodies, potentially hindering the germinal center reaction and B cell maturation [32], as proposed by Yi-Chou Hou and colleagues in their discussion of the effectiveness of COVID-19 vaccines in kidney-transplanted patients [33]. In our cohort, those individuals exhibiting a lower immune response and a lack of neutralizing antibody activity were precisely the patients who, due to post-transplant complications such as graft-versus-host disease, had to prolong their immunosuppressive treatment. Interestingly, in our study, we observed that about 87% of the allo-HCT recipients vaccinated with the mRNA-1273 vaccine, encoding the Wuhan-Hu-1 spike protein, also presented IgG capable of binding and blocking the interaction between the Omicron BA1 RBD and ACE2 receptor. The Omicron variant represents the most divergent strain observed in substantial numbers thus far in the pandemic, giving rise to concerns related to heightened transmissibility, diminished vaccine efficiency, and an elevated risk of reinfection [34]. Achieving long-term immunogenicity through vaccination presents a specific challenge in patients with blood malignancies who have undergone HCT. These investigations contribute to defining the most effective approach to ensure long-term immunity in this vulnerable group. 

This study has strengths and limitations. The strengths include the profiling of the spike-specific memory B cells induced by SARS-CoV-2 vaccination and their persistence from baseline up to one month after the fourth mRNA-1273 vaccine administration in allo-HCT recipients. The major limitation of our study is the relatively small sample size. Another potential weakness of the study is the different time intervals at which the booster vaccine doses were administered in the allo-HCT recipients compared to the HCs.

In summary, this study provides evidence that repeated doses of mRNA-1273 vaccine considerably enhance SARS-CoV-2 specific memory B cell and antibody responses in allo-HCT recipients, underlining the importance of further vaccine doses for allo-HCT recipients who may have achieved a limited response to the primary series of SARS-CoV-2 vaccination.

## Figures and Tables

**Table 1:** Characteristics of the subjects.

	Allo-HCT (n = 56)	Healthy Controls (HCs, n = 34)	*p*
**General variables**			
Age ^a^	53 (45–61)	53 (45–62)	0.824
Male	33 (58.9%)	20 (58.8%)	>0.999
Female	23 (41.1%)	14 (41.2%)	
**Transplantation**			
Time post allo-HCT (years) ^b^	6.4 (0.2–18.9)	-	
**Disease before allo-HCT**			
Acute lymphoblastic leukemia	10 (17.9%)	-	
Acute myeloid leukemia	29 (51.8%)	-	
Chronic lymphocytic leukemia	4 (7.1%)	-	
Myelodysplastic syndromes	3 (5.4%)	-	
Myelofibrosis	1 (1.8%)	-	
Multiple myeloma	3 (5.4%)	-	
Other blood malignancies	6 (10.6%)	-	
**Comorbidities**			
Chronic graft versus host disease	5 (8.9%)	-	
Diabetes	4 (7.1%)	-	
Other comorbidities	14 (25.0%)	-	
**Therapy**			
Immunosuppressive therapy	11 (19.6%)	-	
Other therapies	1 (1.8%)	-	

Notes: values are expressed as n (%), except for ^a^ mean (range). ^b^ years were calculated at first dose administration date. Other comorbidities: hyperthyroidism, celiac disease, severe obesity, Sjogren’s syndrome. Other therapies: methimazole. Abbreviations: -, not applicable.

## Data Availability

Due to ethical and privacy restrictions, the data presented in this study are available upon reasonable request to the corresponding author.

## Supplementary Materials

The following supporting information can be downloaded at: https://www.mdpi.com/article/10.3390/vaccines12040368/s1, Table S1: Variables associated with log-transformed IgG titres at +30 v3 and at +30 v4 (Multiple regression analysis); Figure S1: Spike-specific IgG production against SARS-CoV-2 in healthy controls.
