# Supplementary material for: Spike-Specific Memory B Cell Response in Hematopoietic Cell Transplantation Recipients following Multiple mRNA-1273 Vaccinations: A Longitudinal Observational Study
Source: Vaccines (Basel). 2024 Mar 29;12(4):368. doi: 10.3390/vaccines12040368 (PMC11054563; doi:10.3390/vaccines12040368)
Supplement: Supplementary file 1 [file vaccines-12-00368-s001.zip › vaccines-2859221-supplementary.pdf]

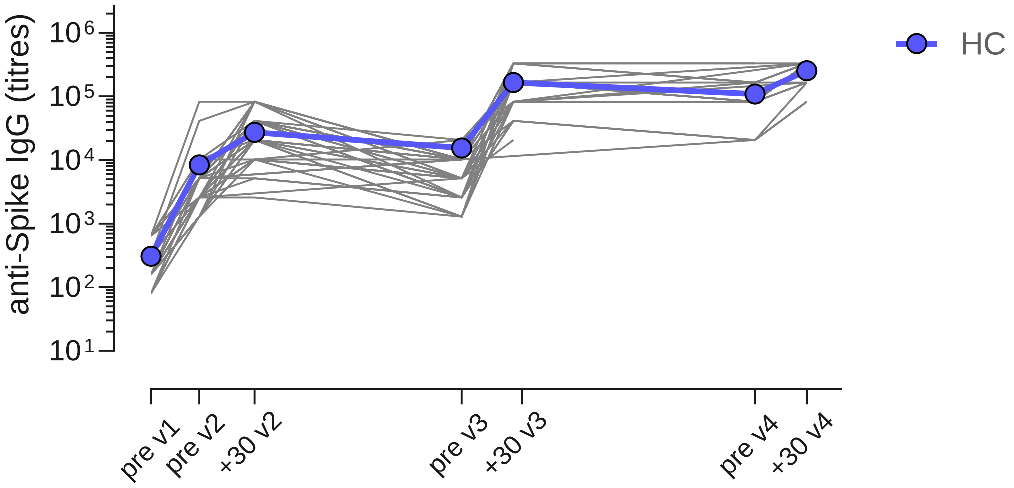

Figure S1. Spike-specific IgG production against SARS-CoV-2 in healthy controls. The anti-spike IgG titers were assessed over time using ELISA, with sampling at baseline (prior to the first dose, pre v1), before the second dose (pre v2), 30 days after the second dose (+30 v2), before the third dose (pre v3), 30 days after the third dose (+30 v3), before the fourth dose (pre v4), and 30 days after the fourth dose (+30 v4). Data are reported in gray for individual subject, and in blue for their mean.

Supplementary Table S1: Variables associated with log-transformed IgG titres at +30 v3 and at +30 v4 (Multiple regression analysis)

	**+30 v3**		**+30 v4**	
**Variable**	**Regression coefficient (95% CI)**	***P***	**Regression coefficient (95% CI)**	***P***
Age	-0.0221 (-0.0776 to 0.0334)	0.418	0.0228 (-0.0396 to 0.0852)	0.456
Gender (male)	0.616 (-0.596 to 1.83)	0.304	0.435 (-0.927 to 1.80)	0.514
Years post transplant	-0.127 (-0.323 to 0.0693)	0.194	-0.184 (-0.405 to 0.0361)	0.097
Immunosuppressive therapy (0/1)	-1.56 (-3.07 to -0.0427)	**0.044**	-0.730 (-2.43 to -0.971)	0.383
GVHD (0/1)	-1.30 (-3.22 to 0.627)	0.176	-0.527 (-2.69 to 1.64)	0.618

Notes: Bold values indicate statistical significance $P \leq 0.05$.
